# Robust $H_{\infty }$-Fuzzy Logic Control for Enhanced Tracking Performance of a Wheeled Mobile Robot in the Presence of Uncertain Nonlinear Perturbations

**DOI:** 10.3390/s20133673

**Published:** 2020-06-30

**Authors:** Nur Syazreen Ahmad

**Affiliations:** School of Electrical and Electronic Engineering, Engineering Campus, Universiti Sains Malaysia, Nibong Tebal, Pulau Pinang 14300, Malaysia; syazreen@usm.my; Tel.: +60-45996014

**Keywords:** robust control, $H_{\infty }$, fuzzy logic, uncertainties, nonlinear, wheeled mobile robot

## Abstract

Motion control involving DC motors requires a closed-loop system with a suitable compensator if tracking performance with high precision is desired. In the case where structural model errors of the motors are more dominating than the effects from noise disturbances, accurate system modelling will be a considerable aid in synthesizing the compensator. The focus of this paper is on enhancing the tracking performance of a wheeled mobile robot (WMR), which is driven by two DC motors that are subject to model parametric uncertainties and uncertain deadzones. For the system at hand, the uncertain nonlinear perturbations are greatly induced by the time-varying power supply, followed by behaviour of motion and speed. In this work, the system is firstly modelled, where correlations between the model parameters and different input datasets as well as voltage supply are obtained via polynomial regressions. A robust $H_{\infty }$-fuzzy logic approach is then proposed to treat the issues due to the aforementioned perturbations. Via the proposed strategy, the $H_{\infty }$ controller and the fuzzy logic (FL) compensator work in tandem to ensure the control law is robust against the model uncertainties. The proposed technique was validated via several real-time experiments, which showed that the speed and path tracking performance can be considerably enhanced when compared with the results via the $H_{\infty }$ controller alone, and the $H_{\infty }$ with the FL compensator, but without the presence of the robust control law.

## 1. Introduction

Motor systems play a foundational role for precise positioning and motion control in robotics and automation, but they are usually subject to nonlinearities, disturbances, as well as sensors’ and environmental noise [1,2]. DC motors in particular are typically vulnerable to the mentioned perturbations; hence, they can vary widely in performance, although they are constructed by the same manufacturer using similar raw materials [3,4]. In this regard, many efforts have been devoted towards modelling DC motors in order to ensure the associated closed-loop systems can operate at varying conditions while preserving the desired performance [5]. Examples include decoupling the linear and nonlinear parts to form the so-called Hammerstein/Wiener structure [6], and employing neural network approach to characterize the nonlinearity [7,8].

Deadzone is one of the most common actuator nonlinearities present at the input of DC motors [9]. This nonlinearity may lead to undesirable effects on closed-loop dynamics and control performance under certain regions. Unlike saturation nonlinearity which is usually characterizable in systems with digital controllers, deadzone is relatively more difficult to model as it can be nonsymmetric and time-varying. A series of control schemes have been developed to alleviate these problems ranging from intelligent [10] to modern robust control approaches [11].

Fuzzy logic (FL) is one type of intelligent control method that has been widely adopted in many feedback control systems due to the nonlinear property of its control gain. A number of control structures have been designed within the fuzzy framework that aim to both attenuate the effects from the nonlinearities and guarantee set point tracking [12], and they are usually referred to as fuzzy logic control (FLC). FLCs can be constructed in different ways, depending on the control objectives and performance specifications. The most common FL approaches are fuzzy proportional-derivative (PD) control [13], fuzzy proportional-integral (PI) control [14], and fuzzy proportional-integral-derivative (PID) control [15,16,17], which are analogous to the structure of the conventional PID controller. While much effort has been directed to PID parameters and control signal tuning from knowledge base and fuzzy inference in order to make the system adaptive to varying conditions and environments [16,18], several studies have also been devoted towards specific FL-based compensation schemes to account for distortions from the nonlinear elements [19,20,21]. In [22], for instance, a two-layered FLC has been proposed to improve the transient performance of existing FLCs, which usually deteriorate when applied to systems with deadzones. A parallel distributed fuzzy model based predictive control was introduced in [23] for a highly nonlinear magnetic suspension system where the stability can be guaranteed via a linear matrix inequality (LMI) approach. Another approach is presented in [24] which employs a FL deadzone compensator combined with sliding mode control that is explicitly designed to alleviate the effects from unknown deadzone nonlinearities and system uncertainties. Apart from nonlinearity compensations, the FL approach has also been adopted to control integral-type plants with parametric uncertainties such as the work presented in [25]. In [26], a combination of linear PID control and FLC to form a cascade loop was introduced to enhance the performance of systems with large delays during real-time implementations.

Although the FL approach may be convenient in the sense that human experience and intuition on the system can be assimilated into the fuzzy rules, a frequently remarked disadvantage of this method is the analysis task related to the controllers’ performance, such as stability and robustness. The modern robust control technique, on the other hand, provides a systematic approach to minimize the effects of perturbations from uncertainties such as varying gains and pole locations within the closed-loop systems by optimizing some performance functions while ensuring stability [27]. $H_{\infty }$-control is one of the methods introduced to deal with systems that are subject to disturbances and errors caused by the uncertainties in the system model [28,29]. By virtue of mixed-sensitivity technique where signals to be minimized or maximized within the closed-loop system are specified a priori, the dynamic compensator can be synthesized by solving the corresponding multi-objective optimization problems. Another principal advantage is the flexibility in the design procedure where the desired structure of the controller and closed-loop poles can be embedded into the optimization problem with convex objective functions, guaranteeing the optimality of the solution.

In the case where structural model errors (i.e., deficiencies or artifacts in the selected model [30,31]) are more dominating than the effects from environments or noise disturbances, accurate system modelling will be highly instrumental in synthesizing the best compensator. Treating the uncertainties in both linear and nonlinear models are equally crucial for enhancing the tracking performance under various circumstances particularly when the system involves multiple DC motors, such as those in wheeled mobile robots (WMRs) [32,33]. Plus, to ensure the compensator is robust against the uncertainties, the source of variations in the model parameters needs to be identified and made auxiliary to the control scheme. Exact sources of errors or uncertainties are often difficult to determine, but influence from other observable parameters, such as electrical energy consumptions [34,35] or nature of motions [36,37] can assist in achieving the same purpose. An interesting study in [38] shows that the electrical power consumptions in a mobile robot can fluctuate under various operations when the same energy supply is used to power the sensor, motion, and control systems.

While much effort has been devoted to energy modelling, power conservation, and optimization techniques [38,39] for precise tracking of WMRs, to the best of the author’s knowledge, there has been to date little or no attention given towards investigating the effects of the power supply variation on the model’s parameters. The main contribution of this work is on enhancing the tracking performance of a WMR, which is driven by two DC motors that are subject to structural model errors that are induced by time-varying power supply as well as behaviour of motions and motor speed. By taking into account the variations of both the model parametric uncertainties and uncertain deadzones into the control law, the WMR is able to self-optimize the tracking performance which is particularly important for movements in remote areas that typically do not allow human interventions in the middle of operations. In this work, the system is firstly modelled where correlations between the model parameters and different input datasets as well as supply voltage are obtained via polynomial regressions. Motivated by hybridization of two control approaches to improve performance [24,26], a robust compensator is proposed by fusing a structured $H_{\infty }$ controller to treat the parametric uncertainties in the linear model, and an FLC to alleviate the effects of the varying deadzones. Via the proposed strategy, the $H_{\infty }$ controller and the FLC work in tandem to ensure the control law is robust against the model uncertainties. The proposed technique was validated via a series of real-time experiments, which showed that the speed and path tracking performance can be considerably enhanced when compared with the results via the $H_{\infty }$ controller alone, and the $H_{\infty }$ with the FLC but without the presence of the robust control law.

The rest of the paper proceeds, as follows; Section 2 describes the problem formulation that includes the description of the motion control system architecture. In Section 3, the main methodology is presented starting from identifying the parameters in the linear and nonlinear blocks together with relations to reference speed and measured power supply, followed by robust controller synthesis to account for the uncertain nonlinear perturbations. Section 4 shows the results that are based on the real-time experiments. The discussions and conclusions are included in the final section.

## 2. Problem Formulation

**Figure 1:**
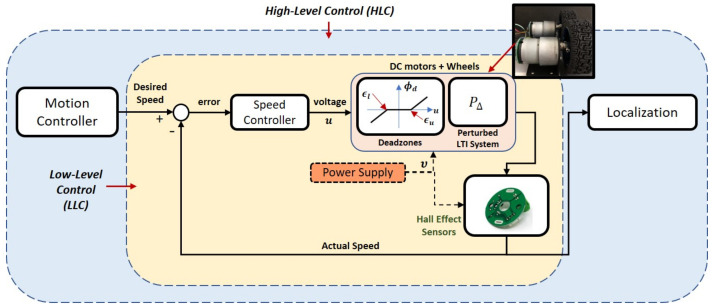
Overview of the motion control system architecture.

An overview of a specific type of motion control system architecture for a WMR is illustrated in Figure 1, which consists of two control layers; high-level control (HLC) and low-level control (LLC). In the LLC layer, the DC motors and the hall effect sensors serve as actuators and speed detectors respectively. The negative feedback is used to ensure that the voltage supplied to the DC motors via the control action *u* can be continuously adjusted through the speed controller, whenever there is a mismatch between the actual speed of the motors and the desired speed generated by the motion controller from the HLC.

Such a control scheme can theoretically suppress the effects of external disturbances and random noise within the LLC layer and result in a high tracking performance. However, in practice, real DC motor models exhibit nonlinear properties and can be time-varying under certain circumstances, which may eventually degrade the performance particularly when the speed controller is only designed based on a specific model of the actuator/plant. The scenario considered in this paper is when the DC motors are subject to model parametric uncertainties as well as uncertain (possibly nonsymmetric) deadzones. As depicted in Figure 1, the deadzone, $\phi_{d}$ (for each motor) is characterized by

(1) $$\begin{array}{c} \phi_{d}\left(u\right)=\left\{\begin{array}{cc} u+\epsilon_{l}\left(\upsilon \right) & \;\mathrm{for}\;\;u<-\epsilon_{l}\left(\upsilon \right)\\ 0\; & \;\mathrm{for}\;-\epsilon_{l}\left(\upsilon \right)\leq u\leq \epsilon_{u}\left(\upsilon \right)\\ u-\epsilon_{u}\left(\upsilon \right) & \;\mathrm{for}\;\;u>\epsilon_{u}\left(\upsilon \right). \end{array}\right. \end{array}$$

with $\epsilon_{l}$ (lower bound) and $\epsilon_{u}$ (upper bound) belong to

(2) $$\Pi_{\phi }=\left\{\epsilon_{i}\left(\upsilon \right)\in R^{+}\;\forall i=l,u\;|\;\epsilon_{i}\left(\upsilon \right)\in \left[\epsilon_{i,min},\epsilon_{i,max}\right]\right\}.$$

where υ is the supply voltage, $\epsilon_{i,min}$ and $\epsilon_{i,max}$ correspond to the minimum and maximum values of $\epsilon_{i}$ respectively. The perturbed LTI system which is represented by $P_{\Delta }$ belongs to a structured set described by

(3) $$\Pi_{p}=\left\{P_{\Delta }=\alpha \left(\upsilon \right)P_{0}\left(s\right):\;P_{0}\in {\mathrm{RH}}_{\infty }\;|\;\alpha \left(\upsilon \right)\in \left[\alpha_{min},\alpha_{max}\right];\;\alpha \in R^{+}\right\}$$

where $P_{0}$ is a first order transfer function, α is the gain uncertainty, $\alpha_{min}$ and $\alpha_{max}$ are the minimum and maximum values of α respectively.

**Figure 2:**
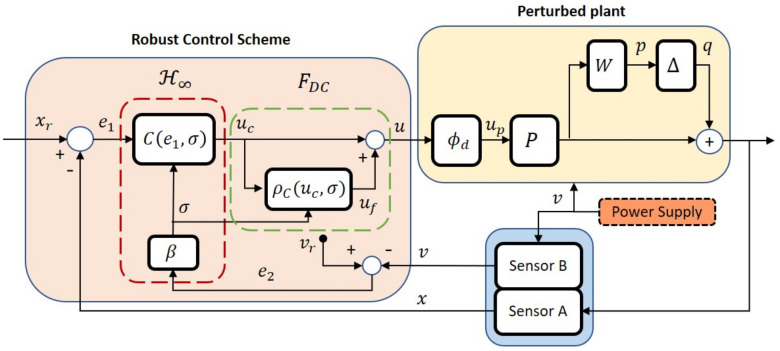
Overall structure of the proposed robust control system in the LLC layer.

In this work, the focus is on robustifying the control law within the LLC layer when the system is subject to the aforementioned structural model errors where the dominant parameters (i.e., $\epsilon_{l},\epsilon_{u},\alpha$) are not only influenced by the speed profile generated by the motion controller, but also by the time-varying voltage from the power supply (i.e., $\upsilon \left(t\right)\in \left(\upsilon_{min},\upsilon_{max}\right)$). Figure 2 shows the overall closed-loop system in the LLC layer that has been restructured with inclusion of a new robust control scheme to account for the uncertain nonlinear perturbations in the plant’s model. The signal $x_{r}$ denotes the desired speed generated from the HLC layer while the signal *x* represents the actual speed of the DC motors measured by Sensor A, which is the hall effect sensor. As the deadzone widths and the plant’s gain are uncertain and dependent on the time-varying power supply, Sensor B is included in the inner loop to measure the voltage driving the DC motors. The main methodology is presented in the following section.

## 3. Methodology

**Figure 3:**
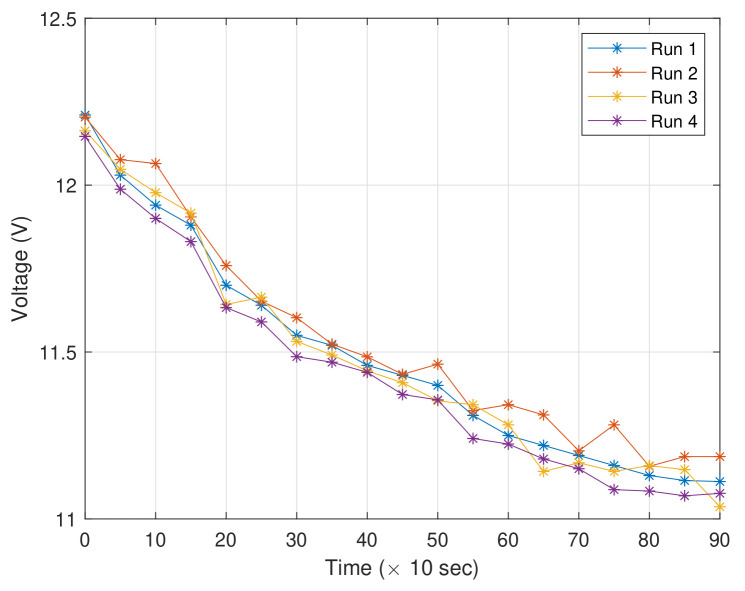
Illustration on variations of the remaining voltage from the power supply under various conditions.

The targeted application of this paper is on precise motion control of a low-cost WMR where a single 3S (3-cell) Lithium-Polymer battery is used as the main supply to power the two DC motors (will be denoted Motor A and Motor B henceforth), the sensors and the control system. It is worth noting that while the whole system can run within the power supply’s operating voltage range (i.e., 11.1 volts to 12.4 volts in this case), the actual voltage to supply the system, υ, can fluctuate within the range due to the nonlinear discharging rate, effects from the ambient temperature, types of motions, as well as load from the sensors. An illustration on the variation of the voltage is depicted in Figure 3 where four samples were recorded; Run 1 and Run 2 represent the cases when the power supply were used to drive the motors at PWM of 50 and 100, while Run 3 and Run 4 represent the same cases as Run 1 and Run 2, but with the sensors attached. The fluctuation may also become higher when the WMR has been running for a longer period (due to the increase in temperature within the WMR).

**Figure 4:**
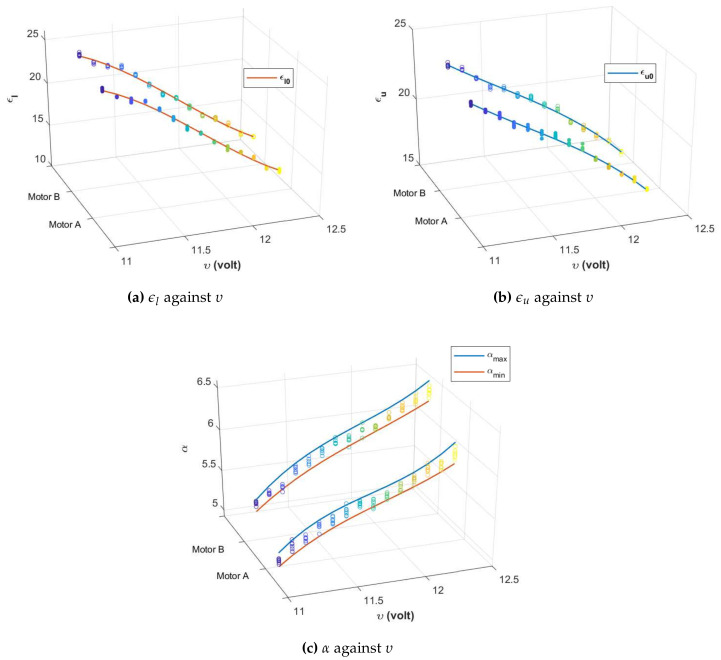
Scatter plots represent the observed values while the curved line plots represent the lines of best fit based on polynomial regressions.

The scatter plots in Figure 4 show the variations of $\epsilon_{l}$, $\epsilon_{u}$ and α against υ from each DC motor that were obtained when ten different input datasets as shown in Table 1 were injected to the plant during the black box identification process (using the MATLAB System Identification Toolbox), with υ varied from $\upsilon_{min}=11.1$ volts to $\upsilon_{max}=12.4$ volts at a step of 0.1 volt. The choice of frequency and amplitude was selected based on motion constraints of the WMR used in this work.

Based on Figure 4a,b, the values of $\epsilon_{l}$ ($\epsilon_{u}$) drop from approximately 24.4 (23.4) to 12.5 (15.0) when υ is increased from 11.1 to 12.4 volts, whereas the values only vary in the range of 1.0 for different inputs. Hence, it can be concluded that the effects of υ on $\epsilon_{l}$ and $\epsilon_{u}$ is more significant than the effects from variations of the input datasets. As the correlations between $\epsilon_{l}$ ($\epsilon_{u}$) and υ will be auxiliary to the robust control scheme, a polynomial regression is used to generate the line of best fit. This method yields

(4) $$\begin{array}{c} \epsilon_{l0}\left(\upsilon \right)=d_{l1}\upsilon^{3}+d_{l2}\upsilon^{2}+d_{l3}\upsilon^{2}+d_{l4};\;\mathrm{and}\;\epsilon_{u0}\left(\upsilon \right)=d_{u1}\upsilon^{3}+d_{u2}\upsilon^{2}+d_{u3}\upsilon^{2}+d_{u4} \end{array}$$

where

$$\begin{array}{c} \left(d_{l1},d_{l2},d_{l3},d_{l4}\right)=\left(3.4,-120.5,1414.2,-5472\right)\\ \left(d_{u1},d_{u2},d_{u3},d_{u4}\right)=\left(-1.5,50.4,-584.6,2302\right) \end{array}$$

for Motor A, and

$$\begin{array}{c} \left(d_{l1},d_{l2},d_{l3},d_{l4}\right)=\left(3.3,-115.9,1360,-525.7\right)\\ \left(d_{u1},d_{u2},d_{u3},d_{u4}\right)=\left(-1.4,48.8,-564.4,2219\right) \end{array}$$

for Motor B. These are represented by the curved line plots in Figure 4a,b, respectively. With regard to Figure 4c, the change of input datasets leads to a relatively larger variation in the value of α as compared to the observations from $\epsilon_{l}$ and $\epsilon_{u}$. Hence, a regression technique is used to formulate the correlation between υ and both the lower and upper bounds of α. The lines of best fit are shown in Figure 4c with parameters as follows:

(5) $$\begin{array}{c} \alpha_{max}\left(\upsilon \right)=m_{1}\upsilon^{3}+m_{2}\upsilon^{2}+m_{3}\upsilon^{2}+m_{4};\;\mathrm{and}\;\alpha_{min}\left(\upsilon \right)=n_{1}\upsilon^{3}+n_{2}\upsilon^{2}+n_{3}\upsilon^{2}+n_{4}, \end{array}$$

where

(6) $$\left\{\begin{array}{cc} \left(m_{1},m_{2},m_{3},m_{4}\right) & =(0.3406,-12.0724,143.3404,-563.96)\\ \left(n_{1},n_{2},n_{3},n_{4}\right) & =(0.3318,-11.8718,142.2345,-564.53) \end{array}\right.$$

for Motor A, and

(7) $$\left\{\begin{array}{cc} \left(m_{1},m_{2},m_{3},m_{4}\right) & =(0.4294,-15.2751,181.8164,-717.882)\\ \left(n_{1},n_{2},n_{3},n_{4}\right) & =(0.5186,-18.4920,220.4168,-872.165) \end{array}\right.$$

for Motor B. The strategies to robustify the control law for treating the uncertainties and the power-supply-induced errors are detailed in the succeeding subsections.

### 3.1. Robust Control Strategy for Treating $\phi_{d}$

Based on the regression technique in the previous subsection, the parameters $\epsilon_{u}$ and $\epsilon_{l}$ of the deadzone nonlinearity, $\phi_{d}$, from Equation 4 can be replaced with $\epsilon_{u0}$ and $\epsilon_{l0}$, respectively. Here, a FLC approach via Takagi–Sugeno (TS) system with Standard Additive Model (SAM) scheme is proposed to compensate for the undesirable effects of the deadzone. In this scheme, the fuzzy system acts as a global fuzzy associative memory that stores *N* fuzzy rules, in which the *j*-th rule is of the form “If $x=A_{j}$ then $y=B_{j}$”, where *x* and *y* are, respectively, its input and output. The crisp value of the output, F:R→R, can then be mathematically expressed as

(8) $$F\left(x\right)=\sum_{j=1}^{N}w_{j}\Omega_{j}\left(x\right)B_{j}$$

where $\Omega_{j}:R\to \left[0,1\right]$ is the membership function which states the degree to which *x* belongs to the “if” part set $A_{j}$, $w_{j}\in R$ is the rule weight, and $B_{j}$ is the control representative value of $\Omega_{j}$.

With reference to Figure 2, the $F_{DC}$ segment represents the compensation scheme for the deadzone using the FLC approach with

(9) $$u=u_{c}+u_{f};$$

where $u_{c}$ is the output from the linear controller *C* (which will be described in the next subsection) and $u_{f}$ is the output from the fuzzy controller $\rho_{c}$. The following proposition shows the method to eliminate the effects of the aforementioned deadzone.

**Proposition** **1.** *Consider the deadzone $\phi_{d}$, as described in Equation (1), with bounds $\left(\epsilon_{l},\epsilon_{u}\right)=\left(\epsilon_{l0},\epsilon_{u0}\right)$ where $\epsilon_{l0}$ and $\epsilon_{u0}$ relate to υ as in Equation (4). Define the membership function*

(10) $$\begin{array}{cc} \Omega_{p}\left(u_{c}\right) & =\left\{\begin{array}{cc} 0 & \;\mathrm{for}\;u_{c}<0\\ 1 & \;\mathrm{for}\;u_{c}\geq 0. \end{array}\right. \end{array}$$

(11) $$\begin{array}{cc} \Omega_{n}\left(u_{c}\right) & =\left\{\begin{array}{cc} 1 & \;\mathrm{for}\;u_{c}<0\\ 0 & \;\mathrm{for}\;u_{c}\geq 0. \end{array}\right. \end{array}$$

*where $u_{c}$ is the input to $\rho_{c}$; the fuzzy logic basis function:*

(12) $$\Omega \left(u_{c}\right)={\left[\begin{array}{cc} \Omega_{p}\left(u_{c}\right) & \Omega_{c}\left(u_{c}\right) \end{array}\right]}^{T};$$

*and the control representative value of $\Omega (u_{c})$:*

(13) $$\hat{\epsilon }\left(\upsilon \right)={\left[\begin{array}{cc} \epsilon_{u0}\left(\upsilon \right) & -\epsilon_{l0}\left(\upsilon \right) \end{array}\right]}^{T}.$$

*Based on the SAM scheme presented in Equation (8), the output of $\rho_{c}$ simplifies to*

(14) $$u_{f}=\rho_{c}\left(u_{c},\upsilon \right)=\hat{\epsilon }{\left(\upsilon \right)}^{T}\Omega \left(u_{c}\right).$$

*If u as expressed in Equation (9) becomes the input to $\phi_{d}$, then the output of $\phi_{d}$, i.e., $u_{p}$ will be*

(15) $$u_{p}=u_{c}$$

**Proof.**  Due to the properties of $\Omega_{p}\left(u_{c}\right)$ and $\Omega_{c}\left(u_{c}\right)$, we have $\Omega_{p}\left(u_{c}\right)=1-\Omega_{c}\left(u_{c}\right)$. Therefore Equations (12)–(14) reduce to:

(16) $$\begin{array}{cc} u_{f}=\rho_{c}\left(u_{c},\upsilon \right) & =\left\{\begin{array}{cc} \epsilon_{u0}\left(\upsilon \right) & \;\mathrm{if}\;u_{c}\geq 0\\ -\epsilon_{l0}\left(\upsilon \right) & \;\mathrm{if}\;u_{c}<0. \end{array}\right. \end{array}$$

Accordingly, the input to $\phi_{d}$ will be

(17) $$\begin{array}{c} u=u_{c}+u_{f}=\left\{\begin{array}{cc} u_{c}+\epsilon_{u0}\left(\upsilon \right) & \;\mathrm{if}\;u_{c}\geq 0\\ u_{c}-\epsilon_{l0}\left(\upsilon \right) & \;\mathrm{if}\;u_{c}<0. \end{array}\right. \end{array}$$

which then gives

(18) $$\begin{array}{c} u_{p}=\phi_{d}\left(u_{c}+u_{f}\right)=\left\{\begin{array}{cc} \left[u_{c}+\epsilon_{u0}\left(\upsilon \right)\right]-\epsilon_{u0}\left(\upsilon \right) & \;\mathrm{if}\;u_{c}\geq 0\\ \left[u_{c}-\epsilon_{u0}\left(\upsilon \right)\right]+\epsilon_{l0}\left(\upsilon \right) & \;\mathrm{if}\;u_{c}<0. \end{array}\right. \end{array}$$

Note that *u* will never be within the deadzone bounds $\left[-\epsilon_{l0},\epsilon_{u0}\right]$. Hence Equation (18) is also equivalent to Equation (15). □

The result presented in Proposition 1 shows that the effects of the deadzone can be can nullified with proper parameter tuning in the fuzzy controller $\rho_{c}$. This will indirectly minimize the control effort from the controller $H_{\infty }$, which will be presented in the next subsection.

### 3.2. Robust Control Strategy for Treating $P_{\Delta }$

With reference to Figure 2, and assuming the proposed $\rho_{c}$ in the preceding subsection cancels out the effects of $\phi_{d}$, the input to the plant simplifies to $u_{p}=u_{c}$ as in Proposition 1. Hence, the remaining issue is the effects of uncertain perturbations in $P_{\Delta }$. The following lemma restructures the perturbed plant, $P_{\Delta }$, into an equivalent form suitable for robust control framework.

**Lemma** **1.** *Consider the real (perturbed) plant, $P_{\Delta }\in \Pi_{p}$, as described in Section 2, with $\alpha_{min}$ and $\alpha_{max}$ expressed as in Equation (5). The perturbed plant can also be restructured into*

(19) $$P_{\Delta }=\underset{P}{\underset{︸}{\alpha_{0}\left(\upsilon \right)P_{0}\left(s\right)}}\left(1+\Delta W\left(\upsilon \right)\right)$$

*where P corresponds to the nominal plant, *Δ* is an uncertainty satisfying $\Delta \in {\mathrm{RH}}_{\infty }$ and ${\left||\Delta |\right|}_{\infty }\leq 1$,*

(20) $$\alpha_{0}\left(\upsilon \right)=\frac{1}{2}\left[\left(m_{1}+n_{1}\right)\upsilon^{3}+\left(m_{2}+n_{2}\right)\upsilon^{2}+\left(m_{3}+n_{3}\right)\upsilon^{2}+\left(m_{4}+n_{4}\right)\right];$$

*and*

(21) $$W\left(\upsilon \right)=\frac{\left(m_{1}-n_{1}\right)\upsilon^{3}+\left(m_{2}-n_{2}\right)\upsilon^{2}+\left(m_{3}-n_{3}\right)\upsilon^{2}+\left(m_{4}-n_{4}\right)}{\left(m_{1}+n_{1}\right)\upsilon^{3}+\left(m_{2}+n_{2}\right)\upsilon^{2}+\left(m_{3}+n_{3}\right)\upsilon^{2}+\left(m_{4}+n_{4}\right)}$$

*which is the weight that characterizes the spatial structure of the uncertainty.*

**Proof.**  Let $\alpha_{0}\left(\upsilon \right)$ be the midpoint between $\alpha_{min}\left(\upsilon \right)$ and $\alpha_{max}\left(\upsilon \right)$. It follows that

$$\alpha_{0}\left(\upsilon \right)=\alpha_{min}\left(\upsilon \right)+\frac{\alpha_{max}\left(\upsilon \right)-\alpha_{min}\left(\upsilon \right)}{2}=\frac{\alpha_{min}\left(\upsilon \right)+\alpha_{max}\left(\upsilon \right)}{2}$$

which is also equivalent to Equation (20). Hence, the uncertain parameter α(υ) can also be written as

$$\begin{array}{cc} \alpha (\upsilon )= & \alpha_{0}\left(\upsilon \right)+\left[\frac{\alpha_{max}\left(\upsilon \right)-\alpha_{min}\left(\upsilon \right)}{2}\right]\Delta ,\;\Delta \in \left[-1,1\right]\\ = & \alpha_{0}\left(\upsilon \right)\left[1+\left(\frac{\alpha_{max}\left(\upsilon \right)-\alpha_{min}\left(\upsilon \right)}{\alpha_{min}\left(\upsilon \right)+\alpha_{max}\left(\upsilon \right)}\right)\Delta \right] \end{array}$$

Since

$$\begin{array}{cc} \frac{\alpha_{max}\left(\upsilon \right)-\alpha_{min}\left(\upsilon \right)}{\alpha_{min}\left(\upsilon \right)+\alpha_{max}\left(\upsilon \right)} & =\frac{\left(m_{1}-n_{1}\right)\upsilon^{3}+\left(m_{2}-n_{2}\right)\upsilon^{2}+\left(m_{3}-n_{3}\right)\upsilon^{2}+\left(m_{4}-n_{4}\right)}{\left(m_{1}+n_{1}\right)\upsilon^{3}+\left(m_{2}+n_{2}\right)\upsilon^{2}+\left(m_{3}+n_{3}\right)\upsilon^{2}+\left(m_{4}+n_{4}\right)}\\ \; & =W(\upsilon ), \end{array}$$

one will get

$$\begin{array}{c} \alpha \left(\upsilon \right)=\alpha_{0}\left(\upsilon \right)\left(1+\Delta W\left(\upsilon \right)\right). \end{array}$$

Thus, the perturbed plant transfer function can be reconstructed with a nominal plant transfer function, *P* (i.e., the transfer function when $\alpha \left(\upsilon \right)=\alpha_{0}\left(\upsilon \right)$) with a multiplicative uncertainty as described in Equation (19). □

**Figure 5:**
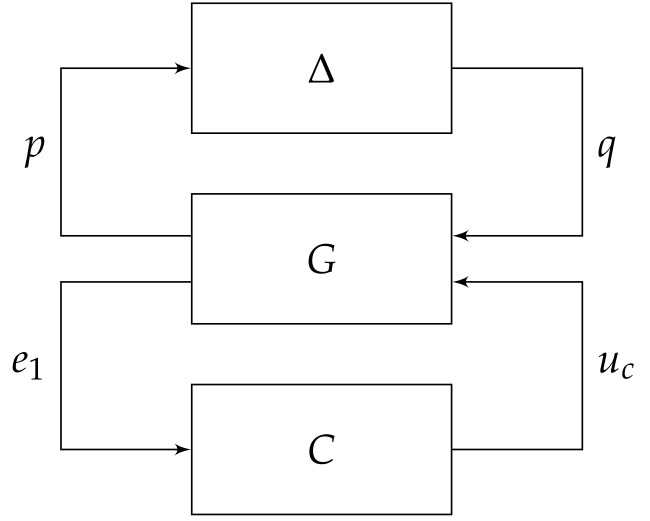
Equivalent closed-loop systems for Figure 2 in general LFT framework where *G* represents the generalized plant, and *C* is the internally stabilizing controller.

By pulling out the uncertainty Δ, Figure 2 can be rearranged into a general linear fractional transformation (LFT) framework, as shown in Figure 5, which can be written as:

(22) $$\left\{\begin{array}{cc} p= & W\left(\upsilon \right)Pu_{c}\\ e_{1}= & -q-Pu_{c}\\ u_{c}= & Ce_{1} \end{array}\right.$$

and $G=\left[\begin{array}{cc} 0 & W(\upsilon )P\\ -I & -P \end{array}\right]$ represents the generalized plant.

For the system at hand, the controller *C* will be synthesized based on the $H_{\infty }$ control objectives, i.e., (i) the feedback interconnection in Figure 5 must internally stable when Δ=0 (i.e., nominal stability); and,

(ii)

(23) $$\;\min_{C}||T_{q\to p}\left(G,C\right)|{|}_{\infty }$$

where $T_{q\to p}\left(G,C\right)$ refers to the transfer function mapping q(s) to p(s), which also corresponds to the lower LFT. Minimizing the $H_{\infty }$-norm of $T_{q\to p}$ as described in (ii) leads to maximization of the size of the allowable uncertainty, ${\left||\Delta |\right|}_{\infty }$, that can be connected to *G* for which the closed-loop system remains internally stable. The following lemma presents the state space representation of the lower LFT for the considered feedback system, with *C* belonging to a class of structured controllers with an integrator.

**Lemma** **2.** *Let $\upsilon_{r}$ represent the nominal operating voltage for the plant, and based on Equations (20) and (21), define*

$$\begin{array}{c} \alpha_{r}=\alpha_{0}\left(\upsilon_{r}\right)\;and\;W_{r}=W\left(\upsilon_{r}\right). \end{array}$$

*Following Lemma 1, consider a nominal plant that is described by*

(24) $$P\left(s\right)=\alpha_{r}P_{0}\left(s\right)∼\left(A_{p},B_{r},C_{p},D_{p}\right)$$

*with $A_{p}\in R^{-}$, $B_{r}=\alpha_{r}B_{p}$, $B_{p}\in R^{+}$, $C_{p}=1$ and $D_{p}=0$; and a structured controller with a state-space given by*

(25) $$\begin{array}{c} C\left(\lambda \right)∼\left[\begin{array}{cc} A_{c} & B_{c}\\ C_{c}\left(\lambda \right) & D_{c}\left(\lambda \right) \end{array}\right],\;\lambda =\left(k_{1},k_{2}\right)\in R^{+} \end{array}$$

*where $A_{c}=0$, $B_{c}=1$, $C_{c}\left(\lambda \right)=k_{1}$ and $D_{c}\left(\lambda \right)=k_{2}$. The state space representation of the lower LFT in (23) can also be written as*

(26) $$\begin{array}{c} T_{q\to p}\left(G,C\left(\lambda \right)\right)∼\left[\begin{array}{cc} A_{1}-B_{1}\Lambda & B_{2}\\ -W_{r}\Lambda & D_{1} \end{array}\right] \end{array}$$

*where*

(27) $$\begin{array}{c} \Lambda =\left[\begin{array}{cc} B_{r}C_{c}\left(\lambda \right) & B_{r}D_{c}\left(\lambda \right) \end{array}\right];\;A_{1}=\left[\begin{array}{cc} 0 & 1\\ 0 & A_{p} \end{array}\right];\;B_{1}=\left[\begin{array}{c} 0\\ 1 \end{array}\right];\;B_{2}=\left[\begin{array}{c} 0\\ 1 \end{array}\right];\;and\;D_{1}=0 \end{array}$$

**Proof.**  From Equation (22),

$$\begin{array}{cc} u_{c}= & \;C\left(\lambda \right)e_{1}\\ = & -C\left(\lambda \right)q-PC\left(\lambda \right)u_{c}\\ \left(1+PC\left(\lambda \right)\right)u_{c}= & -C(\lambda )q\\ u_{c}= & -C\left(\lambda \right){\left(1+PC\left(\lambda \right)\right)}^{-1}q \end{array}$$

Writing *p* in terms of *q*, one will get

$$\begin{array}{c} p=W_{r}Pu_{c}=-W_{r}PC\left(\lambda \right){\left(1+PC\left(\lambda \right)\right)}^{-1}q. \end{array}$$

Hence, $T_{q\to p}\left(G,C\left(\lambda \right)\right)$, which is the transfer function from q(s) to p(s), becomes

$$\begin{array}{c} T_{q\to p}\left(G,C\left(\lambda \right)\right)=\frac{-W_{r}B_{r}D_{c}\left(\lambda \right)s-W_{r}B_{r}C_{c}\left(\lambda \right)}{s^{2}-\left(A_{p}-B_{r}D_{c}\left(\lambda \right)\right)s+B_{r}C_{c}\left(\lambda \right)}. \end{array}$$

Transforming $T_{q\to p}\left(G,C\left(\lambda \right)\right)$ into the controller canonical form, the state space representation of $T_{q\to p}\left(G,C\left(\lambda \right)\right)$ can be written as

(28) $$\begin{array}{c} T_{q\to p}\left(G,C\left(\lambda \right)\right)∼\left[\begin{array}{ccc} 0 & 1 & 0\\ -B_{r}C_{c}\left(\lambda \right) & A_{p}-B_{r}D_{c}\left(\lambda \right) & 1\\ -W_{r}B_{r}C_{c}\left(\lambda \right) & -W_{r}B_{r}D_{c}\left(\lambda \right) & 0 \end{array}\right]. \end{array}$$

Comparing Equations (26) and (27) with Equation (28), it is straightforward that both state space representations are equivalent. □

The controller *C* in Lemma 2 is synthesized based on an embedded assumption that the voltage supplied to the system is at $\upsilon =\upsilon_{r}$. As the operating voltage can vary between $\upsilon_{min}$ and $\upsilon_{max}$, W(υ) and $\alpha_{0}\left(\upsilon \right)$ will be time-varying. From Equations (20) and (21), *W* and $\alpha_{0}$ obtained vary within the range of:

$$-0.1738\leq W\left(\upsilon \right)\leq -0.1694;\;\mathrm{and}\;5.45\leq a_{0}\left(\upsilon \right)\leq 6.52.$$

The difference between lower and upper bounds of *W* is only 0.0074, which implies that the width between $\alpha_{min}$ and $\alpha_{max}$ is almost consistent for all of the values of υ considered. This can also be observed from Figure 4c. The value of $\alpha_{0}$ on the other hand has a relatively larger variation, hence the controller needs to be optimized based on the current value of $\alpha_{0}$ to ensure the desired performance requirements are always satisfied. The following proposition reformulates the $H_{\infty }$ control problem into a search of *C* that is not only meeting the objectives in (i) and (ii) above, but is also self-adaptive to the variations of υ. The result is further presented in the form of LMIs along with pole placement requirements.

**Proposition** **2.** *Consider the nominal plant P and the controller C with structures, as described in Lemma 2. Let $Y=Y^{T}>0\in R^{n\times n}$, and X=ΛY with $\Lambda ,X\in R^{1\times n}$, and define a set of matrices:*

(29) $$\begin{array}{cc} \; & M_{1}\left(Y,X\right)=A_{1}Y+YA_{1}^{T}-B_{1}X-X^{T}B_{1} \end{array}$$

(30) $$\begin{array}{cc} \; & M_{2}\left(Y,X\right)=A_{1}Y-YA_{1}^{T}-B_{1}X+X^{T}B_{1} \end{array}$$

***(a) Optimal control parameter search:** Given that the desired closed-loop poles of the LLC loop lie in the region bounded by $-p_{L}$, $-p_{R}$, and $\phi_{s}$ in the complex s-plane, where $|p_{R}\left|<\right|p_{L}|$ with $p_{L},p_{R}\in R^{+}$, and $\phi_{s}$ is the angle from the negative real axis. If there exist such Y, X and γ∈(0,1] such that the set of LMIs below is feasible,*

(31) $$\begin{array}{cc} \left[\begin{array}{ccc} M_{1}\left(Y,X\right) & B_{2} & -W_{r}X^{T}\\ B_{2}^{T} & -\gamma I & D_{1}\\ -W_{r}X & 0 & -\gamma I \end{array}\right]< & \;0, \end{array}$$

(32) $$\begin{array}{cc} \left[\begin{array}{cc} M_{1}\left(Y,X\right)\sin\phi_{s} & M_{2}\left(Y,X\right)\cos\phi_{s}\\ M_{2}^{T}\left(Y,X\right)\cos\phi_{s} & M_{1}\left(Y,X\right)\sin\phi_{s} \end{array}\right]< & \;0, \end{array}$$

(33) $$\begin{array}{cc} M_{1}\left(Y,X\right)+2p_{R}Y< & \;0, \end{array}$$

(34) $$\begin{array}{cc} M_{1}\left(Y,X\right)+2p_{L}Y> & \;0, \end{array}$$

*the optimal parameter Λ can be obtained as*

(35) $$\Lambda =XY^{-1}.$$

***(b) Self-adaptive controller design:** With reference to Figure 2, we have $e_{2}=\upsilon_{r}-\upsilon$ which denotes the mismatch between the real voltage supplied to the system and the nominal operating voltage. Let*

(36) $$\left[\begin{array}{cc} C_{cr}\left(\lambda \right) & D_{cr}\left(\lambda \right) \end{array}\right]=\Lambda {\left(B_{r}\right)}^{-1}$$

*and define*

(37) $$C_{r}\left(e_{1}\right)∼\left[\begin{array}{cc} A_{c} & B_{c}\\ C_{cr}\left(\lambda \right) & D_{cr}\left(\lambda \right) \end{array}\right].$$

*which is the state-space of C when $e_{2}=0$. The overall structure of C can then be synthesized as follows:*

(38) $$\begin{array}{c} C\left(e_{1},\sigma \right)=\left\{\begin{array}{cc} C_{r}{\left(e_{1}\right)}^{*}\alpha_{r}{\left[\alpha_{0}\left(\sigma \right)\right]}^{-1} & \;for\;\sigma >0\\ C_{r}\left(e_{1}\right)\; & \;for\;\sigma =0 \end{array}\right. \end{array}$$

*where*

(39) $$\begin{array}{c} \sigma =\beta \left(e_{2}\right)=\left\{\begin{array}{cc} \upsilon_{r}-e_{2} & \;for\;e_{2}\neq 0\\ 0\; & \;for\;e_{2}=0 \end{array}\right. \end{array}$$

*and $\alpha_{0}\left(\sigma \right)$ follows from Equation (20).*

**Proof.**  **(a)** From the $H_{\infty }$ control objective in Equation (23), let

(40) $$||T_{q\to p}\left(G,C\right)|{|}_{\infty }<\gamma$$

or equivalently,

(41) $$||W_{r}P\left(j\omega \right)C\left(j\omega \right){\left(I+P\left(j\omega \right)C\left(j\omega \right)\right)}^{-1}|{|}_{\infty }<\gamma \;\forall \omega \in R.$$

Let $T_{q\to p}∼\left(A_{T},B_{T},C_{T},D_{T}\right)$ where $A_{T}\in R^{n\times n}$, $B_{T}\in R^{n\times 1}$, $C_{T}\in R^{1\times n}$ and $D_{T}\in R$. Invoking the bounded real lemma [28], there exist $Q=Q^{T}\in R^{n\times n}$ and γ>0 such that

(42) $$\left[\begin{array}{ccc} A_{T}^{T}Q+QA_{T} & QB_{T} & C_{T}^{T}\\ B_{T}^{T}Q & -\gamma I & D_{T}^{T}\\ C_{T} & D_{T} & -\gamma I \end{array}\right]<0$$

Substituting $\left(A_{T},B_{T},C_{T},D_{T}\right)$ with those in Equations (26) and (27), inequality (42) can be expanded as follows:

(43) $$\left[\begin{array}{ccc} {\left(A_{1}-B_{1}\Lambda \right)}^{T}Q+Q\left(A_{1}-B_{1}\Lambda \right) & QB_{2} & -{\left(W_{r}\Lambda \right)}^{T}\\ B_{2}^{T}Q & -\gamma I & D_{1}^{T}\\ -W_{r}\Lambda & D_{1} & -\gamma I \end{array}\right]<0$$

Applying a congruence transformation with diag (Y,I,I) on Equation (43), where $Y=Y^{T}=Q^{-1}$, one will obtain obtain

(44) $$\left[\begin{array}{ccc} Y{\left(A_{1}-B_{1}\Lambda \right)}^{T}+\left(A_{1}-B_{1}\Lambda \right)Y & B_{2} & -W_{r}{\left(\Lambda Y\right)}^{T}\\ B_{2}^{T} & -\gamma I & D_{1}^{T}\\ -W_{r}\Lambda Y & D_{1} & -\gamma I \end{array}\right]<0$$

which then simplifies to

(45) $$\left[\begin{array}{ccc} YA_{1}^{T}-{\left(B_{1}X\right)}^{T}+A_{1}Y-B_{1}X & B_{2} & -W_{r}X^{T}\\ B_{2}^{T} & -\gamma I & D_{1}^{T}\\ -W_{r}X & D_{1} & -\gamma I \end{array}\right]<0$$

where X=ΛY. Hence, the LMI (31). If Y>0, then $A_{T}$ is Hurwitz which implies the existence of an internally stabilizing controller, $C=C_{r}$, as in Equations (35)–(37). In order to ensure the resulting closed-loop poles are within the region bounded by $-p_{L},-p_{R}$ and $\phi_{s}$ in the complex s-plane, let the state space of the closed-loop transfer function be represented by $\left(A_{T0},B_{T0},C_{T0},D_{T0}\right)$. The following matrix inequalities can then be applied:

(46) $$\begin{array}{cc} \left[\begin{array}{cc} \left(A_{T0}^{T}Q+QA_{T0}\right)\sin\phi_{s} & \left(A_{T0}^{T}Q-QA_{T0}\right)\cos\phi_{s}\\ {\left(A_{T0}^{T}Q-QA_{T0}\right)}^{T}\cos\phi_{s} & \left(A_{T0}^{T}Q+QA_{T0}\right)\sin\phi_{s} \end{array}\right] & <0 \end{array}$$

(47) $$\begin{array}{cc} A_{T0}^{T}Q+QA_{T0}+2p_{L}Q & <0 \end{array}$$

(48) $$\begin{array}{cc} A_{T0}^{T}Q+QA_{T0}+2p_{R}Q & >0 \end{array}$$

Since $A_{T0}=A_{T}$, applying a congruence transformation with diag (Y,I) on inequality (46) and Y on inequalities (47) and (48) gives the LMIs (32)–(34).

**(b)** In the case where $\upsilon \neq \upsilon_{r}$, the nominal plant becomes

(49) $$\hat{P}\left(s\right)=P\left(s\right)\alpha_{r}^{-1}\alpha_{0}\left(\upsilon \right)∼\left(A_{p},{\hat{B}}_{r},C_{p},D_{p}\right)$$

where ${\hat{B}}_{r}=B_{r}\alpha_{r}^{-1}\alpha_{0}\left(\upsilon \right)$. Thus, applying the LMIs (31)–(34) will give

(50) $$\begin{array}{cc} \left[\begin{array}{cc} C_{c}\left(\lambda \right) & D_{c}\left(\lambda \right) \end{array}\right]= & \Lambda {\left({\hat{B}}_{r}\right)}^{-1} \end{array}$$

(51) $$\begin{array}{cc} = & \Lambda {\left(B_{r}\right)}^{-1}\alpha_{r}{\left[\alpha_{0}\left(\upsilon \right)\right]}^{-1} \end{array}$$

(52) $$\begin{array}{cc} = & \left[\begin{array}{cc} C_{cr}\left(\lambda \right) & D_{cr}\left(\lambda \right) \end{array}\right]\alpha_{r}{\left[\alpha_{0}\left(\upsilon \right)\right]}^{-1} \end{array}$$

Hence Equations (38) and (39) are fair. □

**Remark** **1.** *The LMIs in Proposition 2 can be easily computed using convex optimization methods. While it is possible to perform the optimization in real-time in order to search for optimal *Λ* whenever $e_{2}\neq 0$, the method that is presented here will lead to a considerably much faster control action as the linear relationship between $\left(C_{c},D_{c}\right)$ when $\upsilon =\upsilon_{r}$ and those when $\upsilon \neq \upsilon_{r}$ can be calculated offline. Thus, $C(e_{1},\sigma )$ can be computed a priori, which makes it more suitable for real-time implementations.*

**Figure 6:**
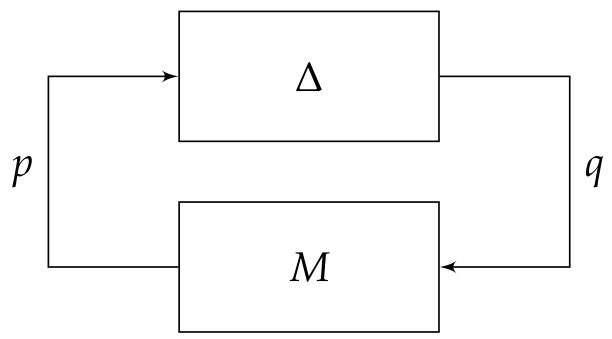
Equivalent *M*-Δ structure with $M=-T_{q\to p}\left(G,C\right)$ for robust stability analysis.

An equivalent *M*-Δ interconnection can be constructed, as illustrated in Figure 6, where *M* contains the lower LFT in order to test whether the interconnected system is robustly stable for all ${\left||\Delta |\right|}_{\infty }\leq 1$.

Assume that the internally stabilizing controller *C* synthesized via Proposition 2 exists. Invoking the small-gain theorem, the closed-loop system will be robustly stable for all $\Delta \in {\mathrm{RH}}_{\infty }$ and ${\left||\Delta |\right|}_{\infty }\leq 1$ if and only if

(53) $$M\in {\mathrm{RH}}_{\infty }{\;\mathrm{and}\;||M||}_{\infty }{\left||\Delta |\right|}_{\infty }<1,$$

or, equivalently,

(54) $$M\in {\mathrm{RH}}_{\infty }{\;\mathrm{and}\;||M||}_{\infty }<1.$$

## 4. Experimental Results

**Figure 7:**
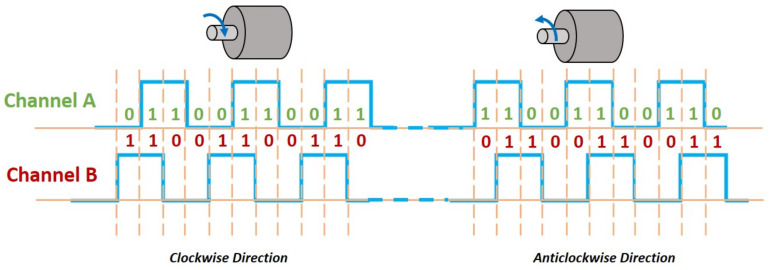
Quadrature encoding with hall effect sensors. Both channels will output square waves that are $90^{\circ }$ out of phase, which allow for both speed and direction to be determined.

In this section, the proposed strategy is validated via two real-time experiments: the first one is designed to analyze the speed tracking performance in the LLC layer and involves only Motor A, whereas the second one is designed to test the path tracking performance of a WMR driven by both Motor A and Motor B. The details on the hardware used are presented in Table 2. The software-hardware interfaces were created using the Simulink Support package for Arduino Hardware (https://uk.mathworks.com/matlabcentral/fileexchange/40312-simulink-support-package-for-arduino-hardware) as well as other custom blocks that are available from the Simulink library. With regard to the hall effect sensor, quadrature encoding technique as depicted in Figure 7 was utilized to allow the wheels’ rotational speeds to be instantly measured with high resolution.

The two methods proposed in Proposition 1 and Proposition 2 will work in tandem to form a robust $H_{\infty }$-fuzzy logic controller to compensate for the uncertain nonlinear perturbations present in the DC motors. For clarity purposes, a superscript *k* in each function/parameter in this section is added to represent the corresponding term for Motor “*k*”. In this work, both motors belong to $\Pi_{p}$ with slightly different dynamics, i.e., $P_{0}^{A}∼\left(-10.23,5.5,1.0,0\right)$, $P_{0}^{B}∼\left(-8.8,5.8,1.0,0\right)$, $\left(\alpha_{min}^{A},\alpha_{max}^{A}\right)$ as described in Equations (5) and (6), and $\left(\alpha_{min}^{B},\alpha_{max}^{B}\right)$ as described in Equations (5) and (7). The nominal operating voltage for both motors, $\upsilon_{r}$, is 11.8 volts. With reference to Lemma 1, we have

(55) $$\begin{array}{cc} \alpha_{0}^{A}\left(\upsilon \right)= & \frac{1}{2}\left(0.6724\upsilon^{3}-23.9442\upsilon^{2}+285.5749\upsilon -1128.5\right)\;\mathrm{and} \end{array}$$

(56) $$\begin{array}{cc} \alpha_{0}^{B}\left(\upsilon \right)= & \frac{1}{2}\left(0.9480\upsilon^{3}-33.7671\upsilon^{2}+402.2332\upsilon -1590.0\right) \end{array}$$

where υ is retrieved via the voltage sensor connected to one of the analog pins of the microcontroller. In particular, the voltage sensor acts as a voltage divider where the output is simply $V_{out}=\frac{R_{2}}{R_{1}+R_{2}}V_{in}$ with a maximum of 5 V. In the Simulink model, the output of the connected analog pin, $V_{out}$, is multiplied with a constant block, $\left({\left(5/1024\right)}^{*}\left(R_{2}+R_{2}\right)/R_{2}\right)$ to give υ, which is similar to $V_{in}$ (the actual supply voltage to the board). Hence, the estimated parameters $\alpha_{0}$ in Equations (55) and (56) can simply be implemented using the available user-defined MATLAB functions.

**Figure 8:**
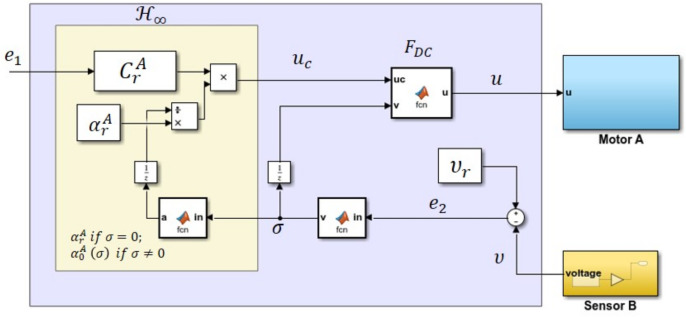
Illustration on the implementation of the proposed controller in the Simulink model.

Supposed it is desired that $\left(p_{L},p_{R}\right)=\left(50,5\right)$, and $\phi_{s}=40^{\circ }$ for each motor response. Applying Proposition 2, one will obtain

(57) $$\begin{array}{c} C^{k}\left(e_{1},\sigma \right)=\left\{\begin{array}{cc} C_{r}^{k}{\left(e_{1}\right)}^{*}\alpha_{r}^{k}{\left[\alpha_{0}^{k}\left(\sigma \right)\right]}^{-1} & \;\mathrm{for}\;\sigma \neq 0\\ C_{r}^{k}\left(e_{1}\right)\; & \;\mathrm{for}\;\sigma =0 \end{array}\right. \end{array}$$

with $C_{r}^{k}\left(e_{1}\right)∼\left(0,1,37.4,4.70\right)$, $\alpha_{r}^{k}=5.5$ when k=A, and $C_{r}^{k}\left(e_{1}\right)∼\left(0,1,34.41,4.86\right)$, $\alpha_{r}^{k}=6.1$ when k=B. An illustration of the implementation of the proposed controller for one of the motors is shown in Figure 8. A unit delay is included for each feedback loop in order to avoid the algebraic loop issue which can cause error during the C code generation.

In order to highlight the improvements introduced by the proposed strategy, the results are also compared against the $H_{\infty }$ controller alone without the FL deadzone compensator, and the $H_{\infty }$ controller with the FL deadzone compensator, but both are without the robust control law (i.e., without the presence of β in Figure 2). The former will be denoted as $H_{\infty }$, the latter as $H_{\infty }$-$F_{DC}$, and the proposed robust method as ${\mathrm{RH}}_{\infty }$-$F_{DC}$. To further demonstrate the robustness of the method against time-varying power supply, the DC motor(s) in each experiment is supplied with a monotonically decreasing voltage using a digital potentiometer, which slowly drops from υ=11.92 volts to υ=11.68 volts during the time of execution.

The performance is then measured in terms of the integral of absolute error, i.e.,

(58) $$E_{IA}=\int_{0}^{t_{L}}\left|e_{\omega }\left(t\right)\right|dt\;\left(\mathrm{rad}\right)$$

where $t_{L}$ is the final time of execution (in seconds), and

(59) $$e_{\omega }\left(t\right)=\omega_{ref}\left(t\right)-\omega \left(t\right)\;\left(\mathrm{rad}/s\right)$$

refers to the instantaneous rotational speed error.

### 4.1. Speed Tracking

**Figure 9:**
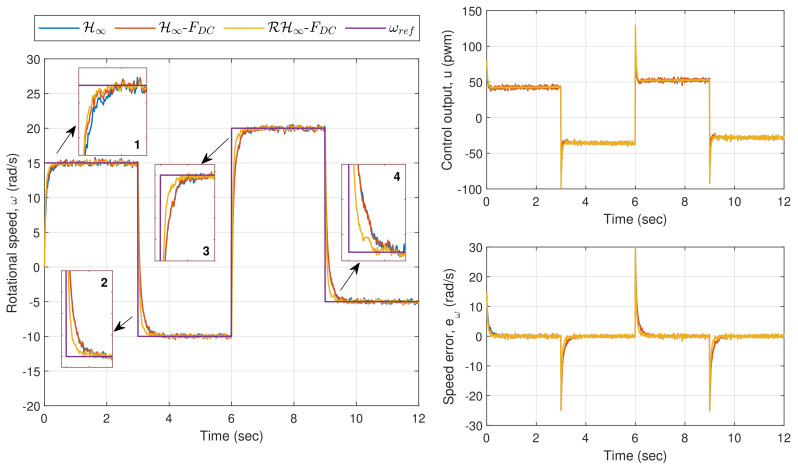
Exp. 1.1: Time responses of Motor A for a changing step input. A faster response is achieved via ${\mathrm{RH}}_{\infty }$-$F_{DC}$.

In the speed tracking performance analysis, three reference speed profiles are tested: (i) a changing step input (Exp. 1.1), (ii) a changing ramp input (Exp. 1.2), and (iii) a sinusoidal wave input (Exp. 1.3). The time responses of Motor A for Exp. 1.1. are presented in Figure 9 where the left plot shows the rotational speed while the right plot shows the corresponding control output (top) and speed error (bottom). It can be observed that the rotational speed via the proposed method settles down to the steady-state value faster than the rest whenever the amplitude is changed, as highlighted in the zoom-in boxes in the left plot. The resulting $E_{IA}$ for $H_{\infty }$, $H_{\infty }$-$F_{DC}$, and ${\mathrm{RH}}_{\infty }$-$F_{DC}$ are 9.5805 rad, 9.2610 rad, and 6.8687 rad, respectively. This shows that the $F_{DC}$ can slightly improve the performance as compared to using the $H_{\infty }$ method alone. However, when both are combined with the inclusion of the robust control law (which gives us ${\mathrm{RH}}_{\infty }$-$F_{DC}$), a significant error reduction can be achieved.

**Figure 10:**
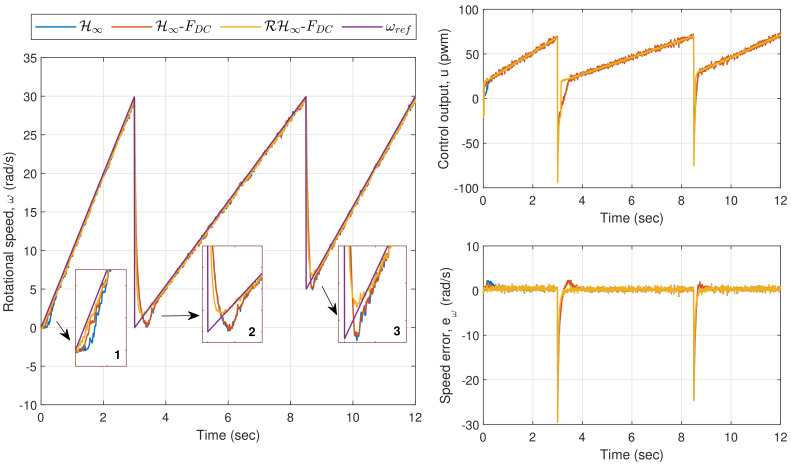
Exp. 1.2: Time responses of Motor A for a changing ramp input. Longer delays and big overshoot can be observed when $H_{\infty }$ and $H_{\infty }$-$F_{DC}$ are applied.

In Exp. 1.2, where the reference speed is a changing ramp input, the corresponding time responses for the motor are shown in Figure 10. From the first zoom-in box of the rotational speed plot, it can be seen that the methods via $H_{\infty }$ and $H_{\infty }$-$F_{DC}$ have introduced longer delays as compared to the proposed method before the speed settles down to its steady-state value. The performance via $H_{\infty }$ and $H_{\infty }$-$F_{DC}$ get worse as can be observed in the second and third zoom-in boxes due to the big overshoot. This is mainly due to the deadzone effects when the control output, *u*, is near to or approaches zero as shown in the top right plot. The effects can also be spotted from the instantaneous errors in the right plot, which result in $E_{IA}$ of 8.4911 rad for $H_{\infty }$, and 8.1733 rad for $H_{\infty }$-$F_{DC}$. The proposed method on the other hand is able to achieve faster response with $E_{IA}$ of 6.8213 rad, which is considerably lower than the other two.

**Figure 11:**
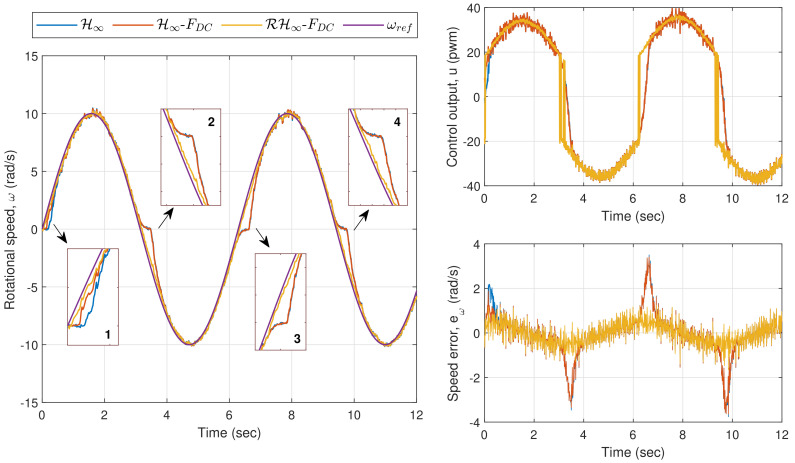
Exp. 1.3: Time responses of Motor A for a sinusoidal wave input. Speed errors are relatively larger via $H_{\infty }$ and $H_{\infty }$-$F_{DC}$ as compared to the error via ${\mathrm{RH}}_{\infty }$-$F_{DC}$.

In the last experiment for this section, which is Exp. 1.3, a sinusoidal wave input with an amplitude of 10 rad/s and a frequency of 1 rad/s is set as the reference speed (the frequency was selected within the frequency range that has been used during the parameter estimation in Section 3). Figure 11 shows the plots for the rotational speed as well as the control output and speed error. A similar trend as in the previous experiment can be observed from the zoom-in boxes of the left plot where larger errors are introduced via the applications of $H_{\infty }$ and $H_{\infty }$-$F_{DC}$, particularly when *u* is near or crosses zero. Nonetheless, the sinusoidal reference speed does not significantly affect the performance when the proposed method is applied, as the controller is able to provide a corrective action particularly when the input to $\rho_{c}$ is driven into the time-varying deadzone area. The corresponding $E_{IA}$ for $H_{\infty }$, $H_{\infty }$-$F_{DC}$, and ${\mathrm{RH}}_{\infty }$-$F_{DC}$ are, respectively, 7.5606 rad, 7.2178 rad and 4.4691 rad. The large difference between the error from the proposed method and the errors from $H_{\infty }$ and $H_{\infty }$-$F_{DC}$ is clearly seen from the bottom right plot of Figure 11.

**Figure 12:**
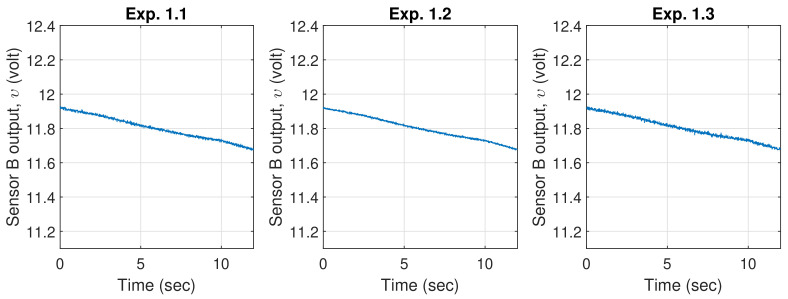
Measured power supply from Sensor B output for each experiment (for validation purposes).

For validation purposes, the output of Sensor B, i.e., υ, is also measured during each experiment. The variation of the signal is depicted in Figure 12.

The numerical results from Experiments 1.1, 1.2, and 1.3 are summarized in Table 3. From the table, it is observed that the proposed strategy consistently results in the lowest error for each experiment. It can also be concluded that, while the presence of $F_{DC}$ can slightly reduce the error, the tracking performance can be significantly enhanced by incorporating the robust control law as proposed in ${\mathrm{RH}}_{\infty }$-$F_{DC}$.

### 4.2. Path Tracking

**Figure 13:**
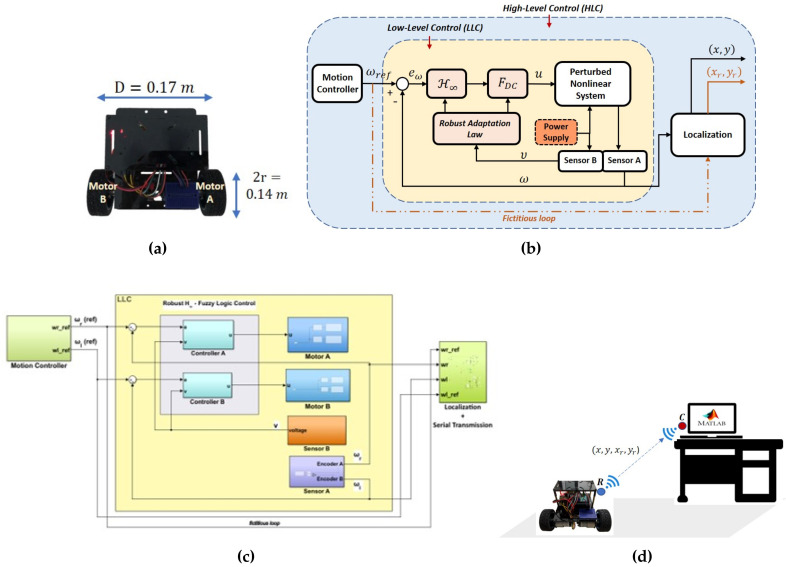
Data acquisition setup for the path tracking experiment with a WMR: (**a**) a sketch (not to scale) of the WMR; (**b**) overview of the proposed model-based control system; (**c**) the Simulink model that has been embedded into the WMR’s microcontroller unit; and, (**d**) a sketch (not to scale) of the wireless data acquisition process from the workstation during the experiment.

For the path tracking performance analysis, it is assumed that the coupling effect between Motor A and Motor B in the WMR is negligibly small. The WMR is of differential type with two identical wheels with radius *r*, positioned at *D* distance apart from each other (refer to Table 2 for the hardware descriptions and Figure 13a for the illustration). Consider [X,Y] as the world’s coordinate, and let $\left(x_{0},y_{0}\right)$ be the center of the shaft, which connects the left and right wheels, and θ be the robot’s heading angle with respect to the positive *X*-axis. In order to simplify the calculation for localization, the new origin is located at $\left(x_{0},y_{0}\right)$, instead of (0,0) of the [X,Y] coordinate. Let $\left(x_{c},y_{c}\right)$ be the current coordinate based on the robot’s frame, denoted as $\left[X_{R},Y_{R}\right]$, and (x,y) be the current coordinate based on the [X,Y] frame. The relation between (x,y), $\left(x_{c},y_{c}\right)$ and $\left(x_{0},y_{0}\right)$ can be written as:

(60) $$\begin{array}{c} \left[\begin{array}{c} x\\ y \end{array}\right]=\left[\begin{array}{cc} \cos\theta & -\sin\theta \\ \sin\theta & \cos\theta \end{array}\right]\left[\begin{array}{c} x_{c}\\ y_{c} \end{array}\right]+\left[\begin{array}{c} x_{0}\\ y_{0} \end{array}\right] \end{array}$$

Define $\omega_{r}$ and $\omega_{l}$ as the rotational speeds of Motor A (at the right wheel) and Motor B (at the left wheel), respectively. For localization puposes where (x,y,θ) needs to be continuously updated with the information from $\omega_{r}$ and $\omega_{l}$ obtained from the hall effect sensors, the movement of the WMR can be decomposed into linear velocity, $\nu_{L}$, and angular velocity, $\nu_{A}$ with respect to the *X*- and *Y*-axes, as follows:

(61) $$\begin{array}{c} \left[\begin{array}{c} \dot{x}\\ \dot{y}\\ \dot{\theta } \end{array}\right]=\left[\begin{array}{cc} \cos\theta & 0\\ \sin\theta & 0\\ 0 & 1 \end{array}\right]\left[\begin{array}{c} \nu_{L}\\ \nu_{A} \end{array}\right] \end{array}$$

where $\nu_{L}=\left(\omega_{r}+\omega_{l}\right)/2$ and $\nu_{A}=\left(\omega_{r}-\omega_{l}\right)r/D$. The current position of the DDR can then be retrieved by integrating (61). Specifically, when the WMR moves forward, (x,y) can be retrieved, as follows:

$$x=\nu_{L}\tau_{s}\cos\theta_{0}+x^{-};\;y=\nu_{L}\tau_{s}\sin\theta_{0}+y^{-};\;\theta =0$$

where $\tau_{s}$ refers to the sampling time and $\left(x^{-},y^{-}\right)$ denotes the previous coordinate of the WMR. When the robot moves to the left or right, a circular arc trajectory will be formed with a radius $r_{arc}=\nu_{L}/\nu_{A}$, and the WMR’s heading angle can be calculated as $\theta =\int_{0}^{\tau_{s}}\nu_{A}dt=\nu_{A}\tau_{s}+\theta^{-}$. The resulting WMR’s position will then be

(62) $$\begin{array}{c} \left[\begin{array}{c} x\\ y\\ \theta \end{array}\right]=\left[\begin{array}{ccc} \cos\theta & -\sin\theta & 0\\ \sin\theta & \cos\theta & 0\\ 0 & 0 & 1 \end{array}\right]\left[\begin{array}{c} r_{arc}\sin\left(\nu_{A}\tau_{s}\right)\\ r_{arc}\left(1-\cos\left(\nu_{A}\tau_{s}\right)\right)\\ \nu_{A}\tau_{s} \end{array}\right]+\left[\begin{array}{c} x^{-}\\ y^{-}\\ \theta^{-} \end{array}\right]. \end{array}$$

In this experiment (i.e., Exp. 2), the desired path is generated based on a speed profile that consists of a mixture of changing ramp and step inputs, which has been designed a priori via the Stateflow toolbox (https://uk.mathworks.com/products/stateflow.html). A sketch of the model-based control system for the WMR is illustrated in Figure 13b, which includes a fictitious loop to generate the desired *x* and *y* positions (i.e., $\left(x_{r},y_{r}\right)$) for performance evaluation purposes. The corresponding Simulink model as depicted in Figure 13c was deployed on the WMR’s microcontroller unit where the data of the speed and positions were serially transmitted via the wireless modules from the WMR to the workstation during the time of execution. As XBee transceivers were used, one module was attached to the WMR and assigned as router (R), while another module was connected to the PC and assigned as coordinator (C), as illustrated in Figure 13d. It is also worth to note that the serial transmission was not part of the main closed-loop operation; hence, the overall motion and control strategy within the WMR was not affected by the wireless transmission. Apart from that, the same procedure as in the first experiment was implemented to demonstrate the performance when the motors are subject to the time-varying power supply.

**Figure 14:**
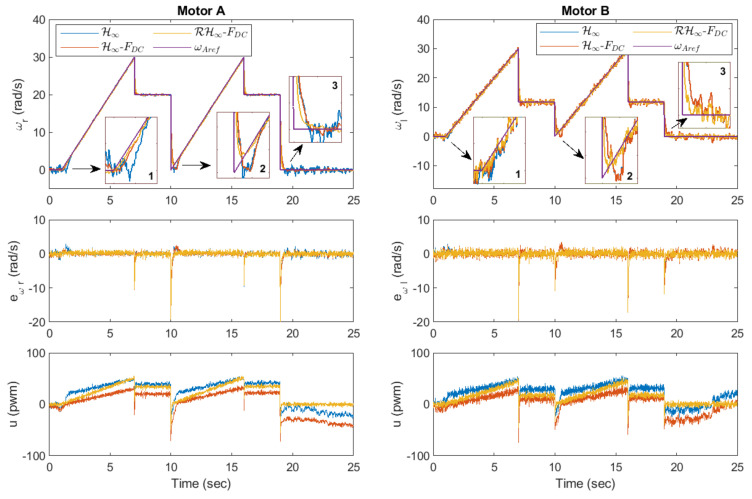
Exp. 2: The left column plots correspond to the time responses for Motor A while the right column plots correspond to the responses for Motor B.

**Figure 15:**
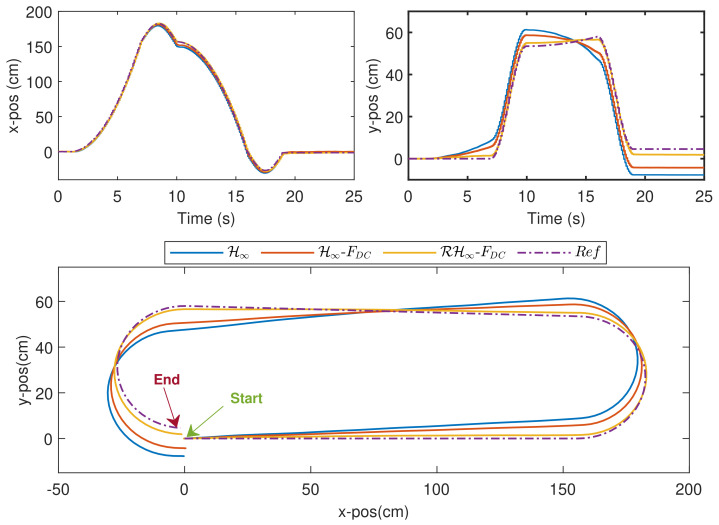
Exp. 2: Trajectories of the WMR in the x-direction (top left), y-direction (top right), and the [X,Y] plane (bottom).

The time responses for both motors are shown in Figure 14, while the trajectories of the WMR are shown in Figure 15. As seen in the zoom-in boxes on the top left plot of Figure 14, the difference of performance for, Motor A between all the three methods is almost similar to that in Exp. 1.3 where ${\mathrm{RH}}_{\infty }$-$F_{DC}$ results in a shorter delay in the beginning, and it does not introduce any overshoot when the control output is close to zero. With regard to Motor B, which has a slightly different dynamic than Motor A, more effects from noise can be seen in the response. Nevertheless, the approach via ${\mathrm{RH}}_{\infty }$-$F_{DC}$ is able to keep the speed closer to the reference value, $\omega_{ref}$.

The position errors propagated from the speed errors can be seen in Figure 15 which clearly shows the deviations of the WMR’s real paths from the reference path via $H_{\infty }$ and $H_{\infty }$-$F_{DC}$ methods. In contrast, with the application of ${\mathrm{RH}}_{\infty }$-$F_{DC}$, the WMR is able to track the reference path with the smallest distance error. In order to evaluate the performance quantitatively, define

(63) $$E_{d}\left(t\right)=\sqrt{{\left(x\left(t\right)-x_{r}\left(t\right)\right)}^{2}+{\left(y\left(t\right)-y_{r}\left(t\right)\right)}^{2}}$$

as the instantaneous distance error with $E_{d,max}=\sup_{t\in [0,t_{L}]}E_{d}\left(t\right)$$\forall t\in [0,t_{end}]$,

(64) $$E_{td}=\int_{0}^{t_{L}}E_{d}\left(t\right)dt$$

as the total position error, and

(65) $$E_{ss}=\sqrt{{\left(x\left(t_{L}\right)-x_{r}\left(t_{L}\right)\right)}^{2}+{\left(y\left(t_{L}\right)-y_{r}\left(t_{L}\right)\right)}^{2}}$$

as the steady-state position error. The experiment was repeated three times and the numerical results with average values were summarized in Table 4. From the table, although the speed errors for $\omega_{r}$ and $\omega_{l}$ are slightly reduced via ${\mathrm{RH}}_{\infty }$-$F_{DC}$, the impact on the path tracking performance can be clearly seen where the corresponding maximum position deviation is only 4.162 cm, which is much smaller than that via $H_{\infty }$ and $H_{\infty }$-$F_{DC}$, where the resulting deviations are, respectively, 13.16 cm and 9.053 cm. The smallest error can also be obtained via the proposed strategy for $E_{td}$ and $E_{ss}$. Hence, when compared with $H_{\infty }$ alone, although $H_{\infty }$-$F_{DC}$ managed to slightly improve the performance as in the speed tracking analysis, a considerable error reduction can only be achieved via ${\mathrm{RH}}_{\infty }$-$F_{DC}$.

### 4.3. Robust Stability Analysis

**Figure 16:**
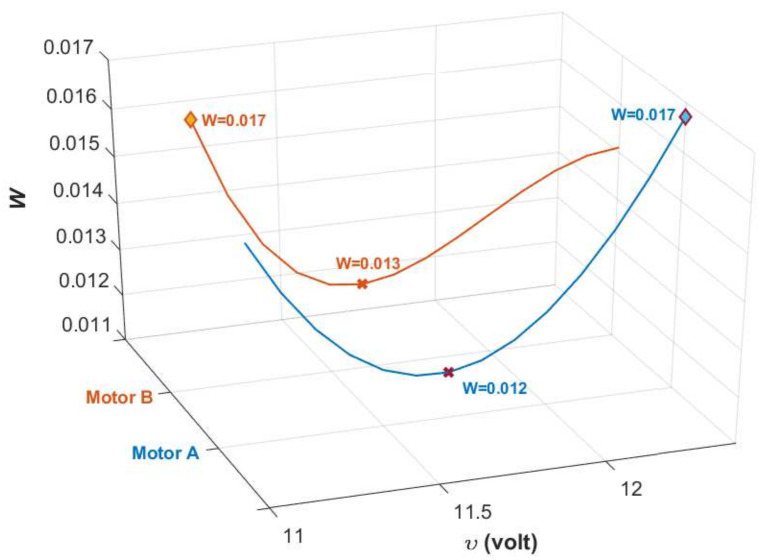
Variation of *W* against υ from $\upsilon_{min}=11.1$ volts until $\upsilon_{max}=12.4$ volts. The plots show that W∈[0.012,0.017] for Motor A and W∈[0.013,0.017] for Motor B.

A plot of *W* against υ for the system considered is depicted in Figure 16, which shows that $\sup_{\upsilon \in [\upsilon_{min},\upsilon_{max}]}W=0.017$. As $M=-WPC{\left(1+PC\right)}^{-1}$ and ${||PC\left(1+PC\right)}^{-1}{\left|\right|}_{\infty }=1$ due to the existence of a closed-loop pole at the origin, then ${\left||W|\right|}_{\infty }<1$ is sufficient to ensure the requirement in (54) is fulfilled.

## 5. Discussions and Conclusions

In this paper, a robust $H_{\infty }$-fuzzy logic compensator is proposed to enhance the speed and tracking performance of a WMR, which is driven by two DC motors that are subject to model parametric uncertainties and varying deadzones. Via polynomial regressions, the correlations between the uncertain parameters in the model and the actual power supplied to the system were formulated. Based on the experimental results, it was shown that when the supply voltage varied, the speed and path tracking errors can be significantly reduced when compared with the results via the $H_{\infty }$ controller alone, and the $H_{\infty }$ with the FLC but without the presence of the robust control law. In certain speed profiles involving ramp and sine wave inputs, longer delays and larger overshoots can be clearly seen without the proposed control strategy. The analysis via the small gain theorem has also shown that the closed-loop system with the designed compensator is robustly stable against the uncertain nonlinear perturbations described in the paper.

While the proposed strategy has been shown to significantly improve the tracking performance, the behaviour of the reference speed considered in this work is limited to changing step and ramp inputs with a certain amount of delay. For further enhancement of tracking strategy, future work may include studying the correlations between the structural model errors with rapid set point changes for scenarios such as collision avoidance strategy for WMRs. However, this may require motion constraints to be considered in the compensator design to ensure other factors, such as impacts from wheel slips or frictions, can be minimized.

## Figures and Tables

**Table 1:** Input datasets for the model’s parameter estimation.

Type	Frequency	Amplitude			Type	Frequency	Amplitude
	(rad/s)	(PWM)				(rad/s)	(PWM)
Step	0	10			Ramp	1.0	80
Step	0.5	20			Ramp	1.5	50
Step	1.0	5			Sine	1.0	50
Step	1.0	15			Sine	1.5	40
Ramp	0	70			Sine	1.0	60

**Table 2:** Hardware descriptions.

Hardware	Descriptions
Microcontroller unit	ATmega2560; Sampling time = 0.01 s
Motors A and B	Brushed type; Load speed: |ω|≤90 rad/s;
	Rated voltage: 12 V; Rated current <200 mA;
	Weight ≈100 g; Motor Driver: TB6612FNG
Hall effect sensors	Quadrature encoding; 390 lines per resolution
Voltage sensor	Input voltage range, $V_{in}$ ≈0.02 to 25 V
	Output voltage range, $V_{out}$: 0 to 5 V
	(acts as a voltage divider with $R_{1}=30$ kΩ, and $R_{2}=7.5$ kΩ)
Wireless Modules	Digi Xbee-S2C 2.4 GHz RF transceiver modules
>WMR	Azimuth length between wheels (*D*): 0.17 m
	Wheel’s radius (*r*): 0.07 m; Weight ≈1 kg
	Size (w×l×h) ≈ 0.19 m × 0.17 m × 0.11 m

**Table 3:** Integral of absolute errors, $E_{IA}$ (rad), from each method for Experiments 1.1, 1.2, and 1.3. The least error for each row is written in bold.

Experiment	Controller		
	**$H_{\infty }$**	**$H_{\infty }$-$F_{DC}$**	**${\mathrm{RH}}_{\infty }$-$F_{DC}$**
Exp. 1.1	9.5805	9.2610	**6.8687**
Exp. 1.2	8.4911	8.1733	**6.8213**
Exp. 1.3	7.5606	7.2178	**4.4691**
Average	8.5441	8.2174	**6.0530**

**Table 4:** Numerical performance evaluations. Each result is an average of three trials. The least value for each row is written in bold.

Performance Index	Controller		
	**$H_{\infty }$**	**$H_{\infty }$-$F_{DC}$**	**${\mathrm{RH}}_{\infty }$-$F_{DC}$**
$E_{IA}$ for $\omega_{r}$ (rad)	15.08	12.38	**11.50**
$E_{IA}$ for $\omega_{l}$ (rad)	15.80	15.74	**13.01**
$E_{d,max}$ (cm)	13.16	9.053	**4.162**
$E_{td}$ (cm s)	207.1	138.1	**52.19**
$E_{ss}$ (cm)	12.33	9.002	**2.729**

## Notations and Abbreviations

The following notations and abbreviations are used in this manuscript:

**Notations/ Abbreviations**	**Descriptions**
FL, FLC	fuzzy logic, fuzzy logic compensator
LMI	linear matrix inequality
WMR	wheeled mobile robot
LLC	low-level control
HLC	high-level-control
PWM	pulse-width modulation
LFT	linear fractional transformation
G,C	generalized plant, internally stabilizing controller
$C^{A},C^{B}$	compensator designed for Motor A, Motor B
*u*	control action (input to the perturbed plant)
$u_{c},u_{f}$	output to the $H_{\infty }$,FL controller
$\upsilon ,\upsilon_{r},\upsilon_{min},\upsilon_{max}$	supply voltage, nominal operating voltage, minimum, maximum supply voltage
$\phi_{d}$	deadzone nonlinearity
$\epsilon_{i(i=l,u,min,max,l0,u0)}$	deadzone bounds (lower, upper, minimum, maximum, best lower, best upper)
$P_{\Delta },P$	perturbed, nominal plant
α	gain uncertainty of the perturbed plant
$\alpha_{0},\alpha_{min},\alpha_{max}$	midpoint, minimum bound, maximum bound of α
$\Pi_{\phi }$, $\Pi_{p}$	class of the deadzone, perturbed plant
*W* ($W_{A},W_{B}$)	weight that characterizes the spatial structure of the uncertainty (for Motor A, Motor B)
$\omega ,\omega_{ref}$	actual, desired rotational speed
$\omega_{r},\omega_{l}$	rotational speed of Motor A, Motor B
$e_{\omega r},e_{\omega l}$	rotational speed error of Motor A, Motor B
$\left(x,y\right),\left(x_{r},y_{r}\right)$	actual, reference coordinates of the WMR
$\nu_{L},\nu_{A}$	WMR’s linear, angular velocities
$\tau_{s},t_{L}$	sampling time, final time of execution (in seconds)
$E_{IA}$, $E_{d}$, $E_{td}$, $E_{ss}$	integral of absolute error, instantaneous distance error, total position error, steady-state position error
R	fields of real numbers
$R^{+}$	fields of positive real numbers
$R^{-}$	fields of negative real numbers
$R^{n\times n}$	fields of n×n real matrices
